# Role of the MAPKs/TGF-β1/TRAF6 signaling pathway in postoperative atrial fibrillation

**DOI:** 10.1371/journal.pone.0173759

**Published:** 2017-03-21

**Authors:** Daoliang Zhang, Xiaoqing Chen, Qian Wang, Shaohui Wu, Yue Zheng, Xu Liu

**Affiliations:** 1 Department of Cardiology, Shanghai Chest Hospital, Shanghai Jiaotong University, Shanghai, People’s Republic of China; 2 Department of Cardiology, Shanghai First People's Hospital, Shanghai, People’s Republic of China; 3 Department of Cardiology, Shanghai Xinhua Hospital, Shanghai, People’s Republic of China; 4 Department of Cardiac Surgery, Shanghai Chest Hospital, Shanghai Jiaotong University, Shanghai, People’s Republic of China; Centro Cardiologico Monzino, ITALY

## Abstract

**Objectives:**

To explore the relationship between the MAPKs/TGF-β1/TRAF6 signaling pathway and atrial fibrosis in patients with rheumatic heart disease (RHD) and its role in atrial fibrillation (AF) after cardiac surgery on the basis of our previous animal study of the MAPKs/TGF-β1/TRAF6 signaling pathway in atrial fibrosis.

**Methods:**

A total of 57 patients with RHD without a history of AF consented to left atrial biopsy. Histopathology quantified the percentage of fibrosis, and real-time PCR and western blot assessed the mRNA and protein expression of TGF-β1, TRAF6, and connective tissue growth factor (CTGF), respectively. Western blot was also used to measure the protein expression of phosphorylated MAPKs and TGF-β-activated kinase 1 (TAK1). Serum angiotensin II (Ang II) levels were assayed using enzyme-linked immunosorbent assay (ELISA).

**Results:**

Eighteen patients developed AF, whereas 39 remained in sinus rhythm (SR). The severity of atrial fibrosis was significantly higher in patients who developed AF versus those who remained in SR; the mRNA and protein expression of TGF-β1, TRAF6 and CTGF were significantly higher in patients with AF. The protein expression of phosphorylated MAPKs and TAK1 was significantly increased in patients who developed AF compared with the patients who remained in SR. Serum Ang II levels were significantly higher in patients who developed AF versus those who remained in SR.

**Conclusion:**

The MAPKs/TGF-β1/TRAF6 signaling pathway is involved in atrial fibrosis in patients with RHD, which results in the occurrence of AF after cardiac surgery.

## Introduction

Atrial fibrillation (AF) is the most common clinical arrhythmia and results in abnormal hemodynamics and thrombotic events that significantly increase morbidity and mortality [1–3]. Atrial fibrillation (AF) occurs in 30% of patients following cardiac surgery and contributes significantly to postoperative morbidity, mortality, and costs [4,5]. One study demonstrated that post-surgical AF was maintained by left atrial (LA) sources and was associated with pre-operative fibrosis [4]. However, the exact mechanism and signal transduction pathway underlying the occurrence and development of atrial fibrosis are unknown [6].

Our previous study demonstrated that the MAPKs/TGF-β1/TRAF6 signaling pathway was involved in mouse atrial fibroblast proliferation induced by AngII and participated in atrial remodeling [7]. The effect of TRAF6 on atrial structural remodeling is not completely clear, nor is the underlying mechanism of this effect, especially in the study of human atrial specimens. We aim to further elucidate the expression of TRAF6 and related signaling pathways in patients with rheumatic heart disease (RHD) and to explore the relationship between the signaling pathways and atrial fibrosis and the role they play in AF after cardiac surgery, based on analysis of recent studies of atrial structural remodeling.

## Materials and methods

### Patient recruitment

After approval from the ethics committee of Shanghai Chest Hospital, as well as the provision of written informed consent by each patient, from December 2013 to December 2014, a total of 57 patients with rheumatic heart disease following cardiac surgery in Shanghai Chest Hospital were enrolled in this study. All patients were given pre-operative cardiac Doppler ultrasound and coronary angiography, and were monitored by continuous ECG telemetry throughout their hospitalization for the presence of AF. In this study, medical records were reviewed to identify the exclusion criteria: history of AF, hyperthyroidism, sick sinus syndrome, pulmonary fibrosis, chronic pulmonary heart disease, cardiomyopathy, kidney disease, liver cirrhosis and patients undergoing a second heart surgery. Cardiac rhythms were defined by a blinded investigator. In addition, pharmacotherapy was discontinued in all patients 12 hours before surgery. All patients were treated following the Declaration of Helsinki of the World Medical Association.

### Sample collection

Left atrial appendage tissue, which was collected before the initiation of extracorporeal circulation, was sectioned into several blocks. One block was fixed with 10% formalin solution for pathological examination, and the other blocks were stored in liquid nitrogen for real-time PCR and Western Blot analysis.

### Histopathology

Specimens, as previously described, were pathologically examined using HE staining and Masson staining. After Masson staining, 4 sections were randomly selected from each patient for quantification of atrial fibrosis. These sections were observed under a Nikon eclipse 50i microscope, and 5 fields were randomly selected from each section. Myocytes were stained red, and collagen fibers were stained blue. Collagen fibers in the endocardium were determined under 400X magnification. Representative photographs were captured with NIS-Elements F camera system, and the proportion of collagen fibers was quantified with Image-Pro Plus 6.0 analysis. The percentage of fibrotic area was calculated as follows: percentage of fibrotic area = fibrotic area / total area × 100.

### Quantitative real-time PCR

Total RNA was extracted from tissue specimens with TRIzol (Invitrogen, USA) and was used to synthesize single-stranded complementary DNA with a high-capacity complementary DNA reverse transcription kit (Toyobo, JAPAN). Quantitative real time RT-PCR involved the use of gene specific primers (see Table 1 for details) and a SYBR kit (Takara, JAPAN). Glyceraldehyde-3-phosphate dehydrogenase (GAPDH) was used as an internal control. Results were expressed as fold difference for each gene against GAPDH by using the 2^-△△Ct^ method.

**Table 1:** Primer sets for PCR amplification.

Gene	Oligonucleotide primer sequences(5’-3’)
TGF-β1	Forward: TGGCGATACCTCAGCAACC
	Reverse: CTCGTGGATCCACTTCCAG
TRAF6	Forward: TTGTGCTAGTGCCCTCGAGAA
	Reverse: CTGGAGGAAAAACTGGGGTGA
CTGF	Forward: GTTTGGCCCAGACCCAACT
	Reverse: GGAACAGGCGCTCCACTCT
GAPDH	Forward: ATGCCAGTGAGCTTCCCGTCAGC
	Reverse: GGTATCGTGGAAGAACTCATGAC

### Serum Ang II level assay

Serum samples were taken 1 day before the surgery. Serum Ang II levels were measured using enzyme-linked immunosorbent assay kits (Enzo Biochem, USA).

### Western blot assay

Left atrial appendage samples were rinsed in cold PBS, and then placed in a 1.5 ml round-bottom tube. Ice-cold RIPA buffer (Beyotime technology), PMSF (0.5ul/ml) and inhibitor of phosphatase was added at 4°C. Total protein was extracted with Protein Extraction Kit (Thermo Scientific, USA) and protein concentration was measured using the BCA Protein Assay Kit (Thermo Scientific, USA), respectively. Protein samples were separated on 12% SDS-PAGE gels and transferred onto nitrocellulose membranes. After blocking with 5% BSA in tris-buffered saline with tween, the membranes were incubated overnight at 4°C with primary antibodies against TGF-β1, CTGF, TRAF6 (Santa Cruz, USA), extracellular signal-regulated kinase1/2 (ERK1/2), phospho-ERK1/2, P38 MAPK, phospho-P38 MAPK, phospho-c-Jun NH(2)-terminal kinase (JNK), TAK1, phospho-TAK1, GAPDH (Cell Signaling Technology, USA), and JNK (Bioworld, USA). After washing, the membranes were incubated with horseradish peroxidase-conjugated secondary antibody for 2 h at room temperature. Western blots were performed using SuperSignal West Femto Chemiluminescent Substrate (Thermo Scientific, USA) and quantified by scanning densitometry. The ratio of interested protein was normalized to GAPDH and was densitometrically analyzed by Quantity One software (USA).

### Statistical analysis

Student's t test was used to compare continuous variables between two groups, and the data for continuous variables were expressed as the mean ± SEM (standard error of the mean). A Chi-square test was adopted to compare categorical variables between two groups. All statistical analyses were performed using SPSS 19.0. Statistical significance was assumed when p<0.05.

## Results

### Patient variables

Of the 57 patients enrolled, 39 patients remained in SR, and 18 developed new onset postoperative AF. Table 2 presents the clinical characteristics of the enrolled patients.

**Table 2:** Patient variables. ACEI = angiotensin-converting enzyme inhibitor; AT antagonist = angiotensin receptor I antagonist; IVSd = interventricular septum; LA = left atrium; LVEDd = left ventricular end-diastolic diameter; LVEF = left ventricular ejection fraction; LVESd = left ventricular end-systolic diameter; LVPWd = Left Ventricular Posterior Wall Depth; PAP = Pulmonary artery pressure.

	AF After Surgery (n = 18)	SR (n = 39)	p-Value
Sex (male/female)	8/10	21/18	0.51
Age (years)	56.4±2.6	56.0±1.4	0.86
Hypertension	5	8	0.54
Diabetes mellitus	4	7	0.7
LVEF (%)	57.4±1.6	58.3±1.3	0.66
LA (mm)	45.0±2.2	42.9±1.3	0.37
LVEDd (mm)	53.1±1.9	54.4±1.6	0.62
LVESd (mm)	34.4±1.8	34.3±1.6	0.98
IVSd (mm)	9.6±0.4	10.6±0.4	0.08
LVPWd (mm)	9.4±0.3	10.0±0.3	0.21
PAP (mmHg)	39.1±3.5	37.8±2.3	0.76
Medication, n			
Diuretics	5	7	0.4
β-Blockers	4	5	0.37
Digitalis	1	6	0.29
ACEI or AT antagonist	8	9	0.1
Calcium antagonist	3	5	0.7
Aldosterone antagonist	3	4	0.49
Statin	2	2	0.41
Nitrate	4	3	0.12

Values are means ± SEM.

### Histopathological staining and quantitative analysis of fibrotic area

**Fig 1:**
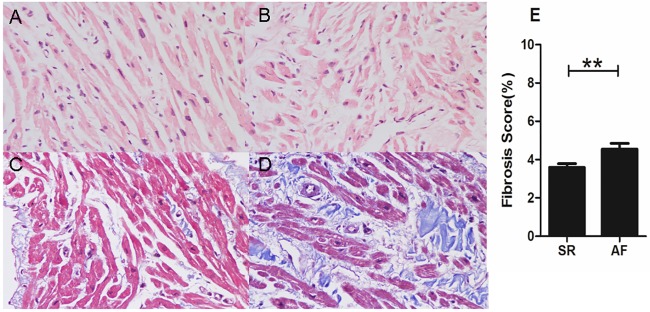
H&E and Masson's trichrome staining of the atrial appendage in SR(A, C) and postoperative AF (B, D) patients (magnification 400×). Compared with SR patients, atrial muscle distribution showed disarray and myocytolysis in postoperative AF patients. There was a significant difference in the fibrosis score between the two groups (E). The bars indicate the means ± SEM. SR = remained in normal sinus rhythm, AF = developed new onset postoperative atrial fibrillation, **p<0.01. All 57 patients are fibrosis scored and represented in panel E.

The results of the left atrial appendage HE staining and Masson staining are illustrated in Fig 1. Compared with the patients who remained in sinus rhythm (Fig 1A), HE staining showed that the atrial muscle cells of atrial appendage tissue in patients with postoperative AF were disordered and hypertrophic, (Fig 1B). Masson staining revealed obvious fibrosis for all patients in the two groups with rheumatic heart disease, but the percentage of fibrosis in the postoperative AF group was more significant (Fig 1C and 1D). The fibrosis grade of the postoperative AF group was significantly higher than that of the sinus rhythm group (p<0.01; Fig 1E), which suggested that there was internal connection between preoperative atrial fibrosis and postoperative AF.

### Real-time PCR

**Fig 2:**
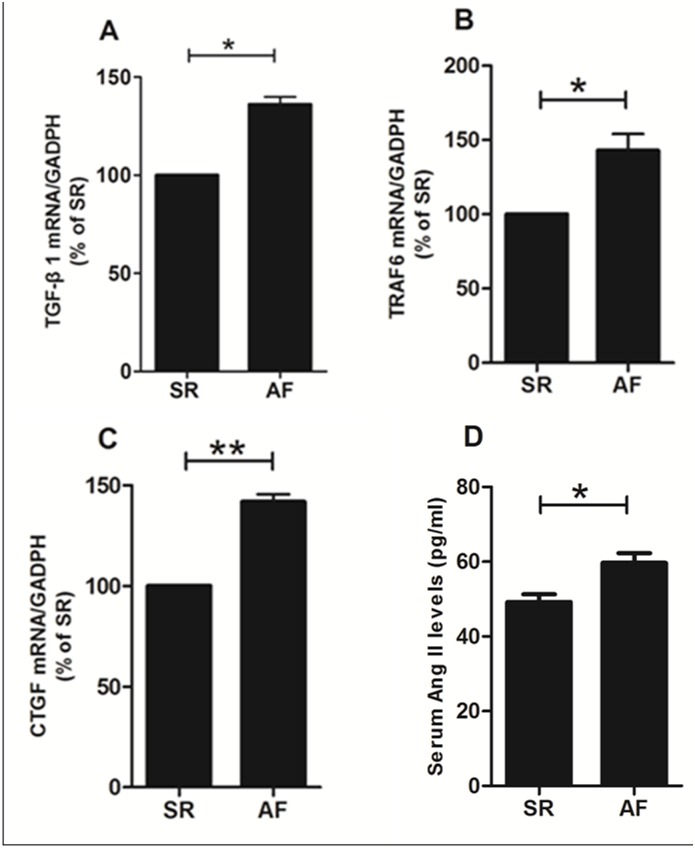
The mRNA expression of TGFβ1 (A), TRAF6 (B) and CTGF (C) in the postoperative AF group was markedly higher than that in the SR group. In each group, tissue samples from 15 random patients were used. All experiments were performed 3 times. Serum Ang II levels were measured in all patients and were significantly higher in patients who developed AF versus those who remained in SR (D). Bars correspond to the mean ± SEM. SR = remained in normal sinus rhythm, AF = developed new onset postoperative atrial fibrillation, **p<0.01, *p<0.05.

Real-time PCR tests have shown that the mRNA expressions of TGFβ1, TRAF6 and CTGF in the postoperative AF group were significantly higher than those in the sinus rhythm group (Fig 2A, 2B and 2C).

### Serum Ang II levels

Serum Ang II levels were significantly higher in patients who developed AF versus those who remained in SR (Fig 2D).

### Western blotting

**Fig 3:**
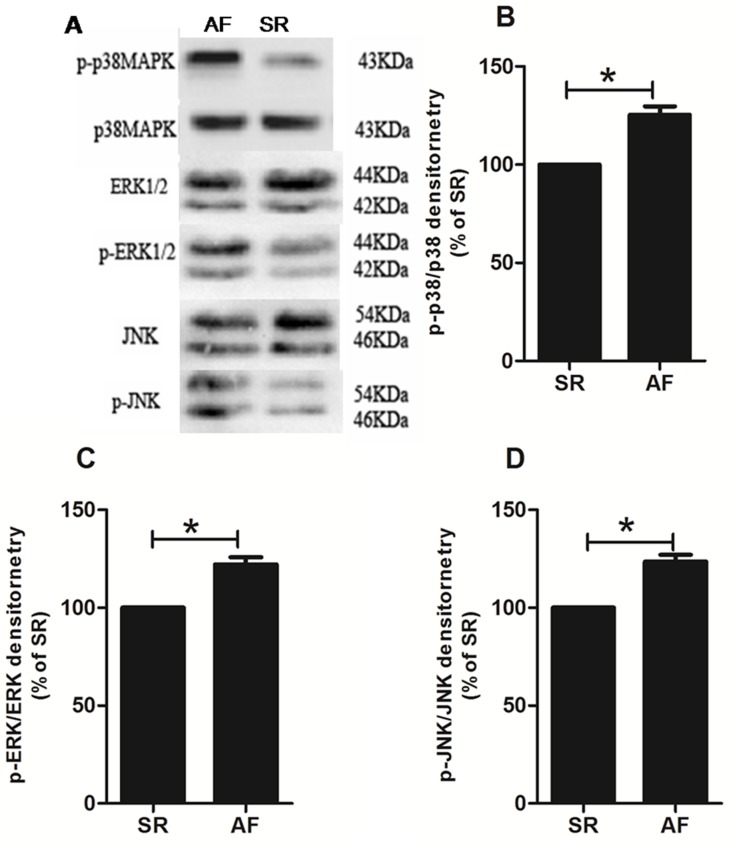
A: Representative western blot of MAPKs. Each band represents one patient with either postoperative AF or SR. The protein expression of phosphorylated P38 MAPKs (B), ERK1/2 (C) and JNK (D) in the postoperative AF group was significantly higher than that in the SR group. Bars correspond to the mean ± SEM. In each group, tissue samples from 15 random patients were used. All experiments were performed 3 times. SR = remained in normal sinus rhythm, AF = developed new onset postoperative atrial fibrillation, *p<0.05.

**Fig 4:**
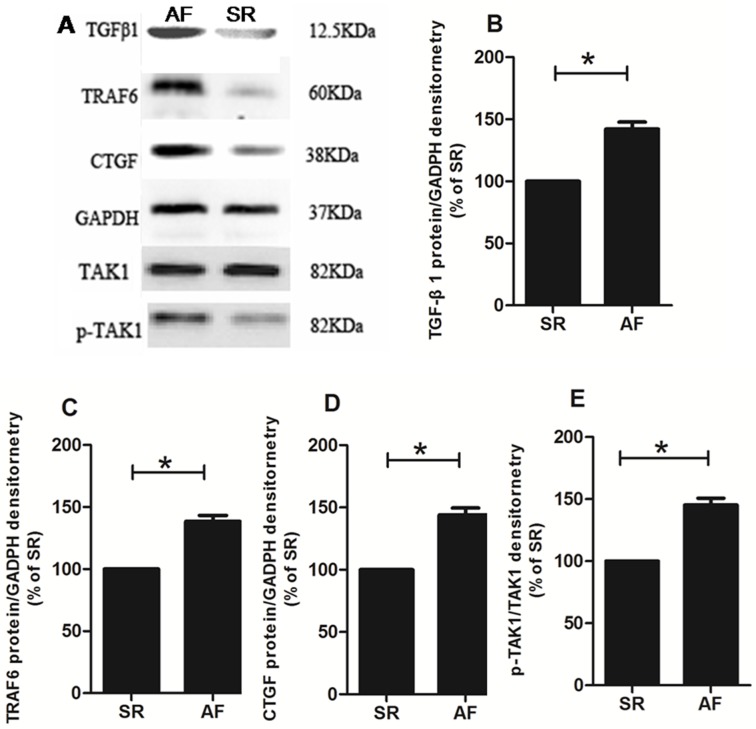
A: Representative western blot of TGFβ1, TRAF6, CTGF and TAK1. Each band represents one patient with either postoperative AF or SR. The protein expression of TGFβ1 (B), TRAF6 (C), CTGF (D) and phosphorylated TAK1 (E) increased significantly in the postoperative AF group. Bars correspond to the mean ± SEM. In each group, tissue samples from 15 random patients were used. All experiments were performed 3 times. SR = remained in normal sinus rhythm, AF = developed new onset postoperative atrial fibrillation, *p<0.05.

Western blotting demonstrated that P38 MAPKs, ERK1/2 and the degree of JNK phosphorylation were higher in the postoperative AF group than in the sinus rhythm group (p<0.05; Fig 3). The protein expressions of TGFβ1, TRAF6 and CTGF and TAK1 phosphorylation in the postoperative AF group were significantly higher than those in the sinus rhythm group (p<0.05; Fig 4). Taken together, these results confirmed that the MAPKs/TGF-β1/TRAF6 signaling pathway is involved in the development of atrial fibrosis in patients with rheumatic heart disease.

## Discussion

**Fig 5:**
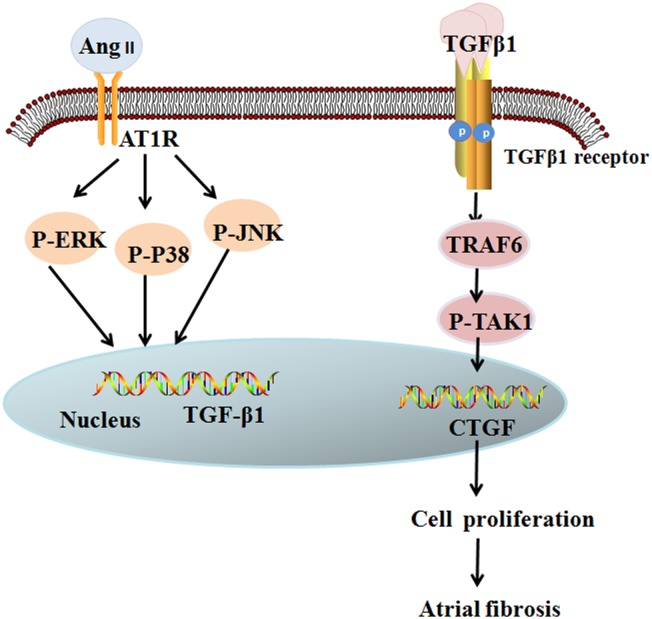
AngII-induced MAPK/TGF-β1/TRAF6/TAK1 signaling pathway in postoperative atrial fibrillation. Binding of Ang II to AT1 receptor induces activation of MAPK, which increases the expression of TGF-β1. Then, paracrine production of TGF-β1 activates the signaling pathway of TRAF6/TAK1. Activation of the above signaling pathway promotes the proliferation of atrial fibroblasts, leading to atrial fibrosis.

In this study we showed that patients without a history of AF who develop postoperative AF have a higher percentage of LA fibrosis,increased expression of TRAF6, higher serum Ang II levels, andchanges in the activities of the MAPKs/TGF-β1/TRAF6pathway. Because our team have previously demonstrated that the MAPKs/TGF-β1/TRAF6 signaling pathway is important in atrial fibrosis development in cultured mice fibroblasts [7], we propose that MAPKs/TGF-β1/TRAF6 is also an important signaling pathway in AngII-induced CTGF expression, which also plays an important role in human atrial structural remodeling (Fig 5).

The exact mechanism and signal transduction pathway underlying atrial muscle fibrosis is unknown [6]. Studies in recent years have noted that the following mechanisms are mainly involved in atrial fibrosis: 1) the renin angiotensin aldosterone system (RAAS), 2) TGF-β1 and 3) inflammation and oxidative stress. Of these, the study of the RAAS system activation mechanism resulting in atrial structural remodeling was relatively thorough.

Ang II is a key link between the RAAS system and atrial fibrosis. In animal models of over-expression of angiotensin converting enzyme (ACE), significantly increased AngII levels resulted in distinct atrial enlargement and atrial fibrosis and eventually caused the occurrence of AF [8] and up-regulation of angiotensin type I receptor (AT1R) expression was found in atrial tissues of patients with AF. [9]. Further studies have found that RAAS inhibitors can inhibit atrial fibrosis and atrial structural remodeling and slow down the occurrence of AF [10,11]. Our finding of increased levels of Ang II in patients who develop postoperative AF also supports this hypothesis.

Studies have discovered that the activation of MAPKs played an important role in atrial structural remodeling. MAPKs, a crucial downstream signaling molecule of AngII, participated in the process of up-regulation of TGF-β1 expression induced by AngII [12–14]which has been considered to be the main mechanism underlying atrial fibrosis. Our previous study found that AngII can improve MAPKs activation, up-regulate expression of TGF-β1 and CTGF, and enhance cell proliferation [7]. This study determined that compared with the patients in sinus rhythm, the degree of MAPKs activation in atrial tissue was significantly increased in patients who developed postoperative AF.

TGF-β1, a key growth factor in fibrosis, can regulate cell proliferation, apoptosis and migration, and extracellular matrix synthesis. It up-regulates the expression of fibronectin and collagen, [15] and the over-expression of TGF-β1 can lead to the occurrence of atrial fibrosis and AF through up-regulation of CTGF expression [16] [12,17]. This study has shown that compared with the patients with sinus rhythm, the mRNA and protein expression of TGF-β1 and CTGF in atrial tissue were significantly elevated in the patients without a history of AF who developed postoperative AF.

Studies have shown that TGF-β1 promoted the occurrence of atrial fibrosis through both downstream Smad signaling [18] and Smad-independent pathways The TRAF6/TAK1 signaling pathway plays a key role in the activation of TGF-β1/non-Smad signaling pathways. The tumor necrosis factor receptor is an important intra-cytoplasmic adaptor molecule; these types of molecules have a TRAF structure domain of approximately 230 amino acids and a characteristic C terminus [19]. At present, TRAF6 is one of 6 types of TRAF molecules identified in mammals; it achieves its biological effect mainly through signal transduction pathways. After TGF-β1 activated the TGF-receptor, it promoted TRAF6 ubiquitination, thereby prompting the combination of TRAF6 with TAK1, and then ubiquitination of TAK1 at lysine 63 and 158, thereby further activating the downstream signaling pathway [20,21]. Studies have reported that the activation of the TRAF6/TAK1 signaling pathway induced by TGF-β1 is involved in apoptosis, inflammation and epithelial mesenchymal transformation [22]. This study determined that the protein expressions of TRAF6 and CTGF and TAK1 phosphorylation in the atrial tissue of patients who develop AF after cardiac surgery with no previous history of AF were significantly higher than those in the sinus rhythm group. Because we previously found that AngII stimulated mouse atrial fibroblasts and that TGF-β1 played a role through the Smad-independent pathway [7], we verified that the TGF-β1/TRAF6 signaling pathway was involved in the development of atrial fibrosis.

However, this study has some limitations. Although all patients had no known history of AF, some could have had prior episodes of asymptomatic AF as pre-operative Holter monitoring was not performed. In addition, we only compared the expression of the related molecules in atrial tissue, but no further extraction and culture of atrial fibroblasts was performed to observe the effect of AngII and TRAF6 on fibroblast proliferation in human atrial tissue and on the MAPKs/TGF-β1/TRAF6 signaling pathway. Therefore, our study verified only that this signaling pathway is altered in the development of atrial fibrosis in patients with no history of AF who developed AF after cardiac surgery.

In summary, our study determined that the occurrence of postoperative AF in patients who have no previous history of AF was correlated with atrial fibrosis and that the MAPKs/TGF-β1/TRAF6 signaling pathway was altered in these patients, which suggests that this pathway is an important signaling pathway in the CTGF expression induced by AngII.

## Supporting information

S1 Dataset Patient variables, fibrosis scores and Ang II levels. (XLSX)

S2 Dataset Results of quantitative real-time PCR. (XLSX)

S3 Dataset Results of western blot assay. (XLSX)
